# Altered Structure and Function of Murine Sclera in Form-Deprivation Myopia

**DOI:** 10.1167/iovs.63.13.13

**Published:** 2022-12-13

**Authors:** Dillon M. Brown, Michael A. Kowalski, Quinn M. Paulus, Jianshi Yu, Praveen Kumar, Maureen A. Kane, Jay M. Patel, C. Ross Ethier, Machelle T. Pardue

**Affiliations:** 1 Department of Biomedical Engineering, Georgia Institute of Technology/Emory University, Atlanta, Georgia, United States; 2 Center for Visual and Neurocognitive Rehabilitation, Atlanta Veterans Affairs Healthcare System, Atlanta, Georgia, United States; 3 Department of Orthopedics, Emory University School of Medicine, Atlanta, Georgia, United States; 4 Department of Pharmaceutical Sciences, University of Maryland School of Pharmacy, Baltimore, Maryland, United States

**Keywords:** myopia, biomechanics, mouse, sclera, glycosaminoglycans

## Abstract

**Purpose:**

The sclera is believed to biomechanically influence eye size, facilitating the excessive axial elongation that occurs during myopigenesis. Here, we test the hypothesis that the sclera will be remodeled and exhibit altered biomechanics in the mouse model of form-deprivation (FD) myopia, accompanied by altered retinoid concentrations, a potential signaling molecule involved in the process.

**Methods:**

Male C57 Bl/6J mice were subjected to unilateral FD (*n* = 44 eyes), leaving the contralateral eye untreated (contra; *n* = 44). Refractive error and ocular biometry were measured in vivo prior to and after 1 or 3 weeks of FD. Ex vivo measurements were made of scleral biomechanical properties (unconfined compression: *n* = 24), scleral sulfated glycosaminoglycan (sGAG) content (dimethylmethylene blue: *n* = 18, and immunohistochemistry: *n* = 22), and ocular all-trans retinoic acid (atRA) concentrations (retina and RPE + choroid + sclera, *n* = 24). Age-matched naïve controls were included for some outcomes (*n* = 32 eyes).

**Results:**

Significant myopia developed after 1 (−2.4 ± 1.1 diopters [D], *P <* 0.001) and 3 weeks of FD (−4.1 ± 0.7 D, *P* = 0.025; mean ± standard deviation). Scleral tensile stiffness and permeability were significantly altered during myopigenesis (stiffness = −31.4 ± 12.7%, *P <* 0.001, and permeability = 224.4 ± 205.5%, *P <* 0.001). Total scleral sGAG content was not measurably altered; however, immunohistochemistry indicated a sustained decrease in chondroitin-4-sulfate and a slower decline in dermatan sulfate. The atRA increased in the retinas of eyes form-deprived for 1 week.

**Conclusions:**

We report that biomechanics and GAG content of the mouse sclera are altered during myopigenesis. All scleral outcomes generally follow the trends found in other species and support a retina-to-sclera signaling cascade underlying mouse myopigenesis.

Myopia is the most common refractive error and cause of visual impairment, predicted to affect over 50% of the population by 2050.^1^ Myopia is typically caused by axial elongation of the eye beyond that necessitated by the eye's optical power (axial myopia)^2^ (i.e. incoming light is focused in front of the retina). This elongation not only disrupts the optics, but also the ocular biomechanical environment, which may explain why excessive axial length is a risk factor for other blinding diseases with biomechanical aspects (e.g. retinal detachment, glaucoma, and optic disk abnormalities).^3^^–^^5^ Thus, preventing myopigenesis is of great interest, yet our understanding of the mechanisms of axial elongation and myopigenesis is lacking.

The physical process of axial elongation appears to be dictated by the biomechanics of the sclera, which in turn are determined by the quantity/types of constituent components and their microstructural arrangement.^6^^,^^7^ Presentation of myopigenic visual stimuli causes significant and rapid scleral remodeling, including changes in sulfated glycosaminoglycan (sGAG) levels and an increase in extensibility (i.e. altered biomechanics).^6^^,^^8^^–^^14^ This relationship between the state of the sclera (structure and biomechanics) and axial length appears to be causal, as supported by both computational modeling^15^ and studies in which the sclera is experimentally stiffened (e.g. using collagen cross-linking agents or other mechanical reinforcement) which result in altered axial length.^16^^,^^17^ However, no proposed mechanisms linking scleral biomechanics to axial length have successfully explained the magnitude and time course of myopigenic axial elongation, indicative of the complexity of this process.

There is great interest in elucidating the mechanism by which myopigenic visual cues are able to influence scleral remodeling.^18^^,^^19^ Significant evidence supports the general idea of a direct retina-to-sclera (retinoscleral) signaling cascade capable of communicating information about magnitude and direction of optical defocus contained in an image.^20^^–^^28^ However, the details behind this signaling cascade remain elusive, especially regarding how the signal is being propagated through the choroid.^18^^,^^19^^,^^29^

Retinoic acid (RA) may be an important signal capable of trans-choroidal signaling within the retinoscleral cascade. All-trans retinoic acid (atRA) concentrations and synthesis in retinal and choroidal tissue have been shown to be correlated to the refractive state and direction of blur, dependent on both tissue and species.^30^^,^^31^ atRA has been previously shown to disproportionately concentrate in the sclera when co-cultured with choroidal tissue,^30^ suggesting the existence of an active transport process, and scleral concentrations of atRA influence the remodeling processes by modulating sGAG and/or synthesis.^32^ Finally, exogenous atRA given to guinea pigs and chickens has been shown to promote axial elongation^30^^,^^33^ and influence scleral remodeling.^34^ However, it is not clear if atRA-induced elongation occurs via the same pathways as myopigenesis.

The mouse model of myopia has great potential to address questions pertaining to mechanisms of myopigenesis. Although the mouse is widely used for studying complex biological mechanisms, it is still not clear how comparable mouse myopigenesis is to other mammalian species and thus to what degree findings in mice may translate.^35^^,^^36^ Additionally, the small size of its eye (approximately 3 mm) introduces significant technical challenges in measuring axial elongation, scleral biomechanics, and quantities of signaling molecules. In the mouse, axial elongation is inconsistently reported to change with myopigenic cues,^36^ and neither scleral GAGs nor biomechanics have been studied in respect to myopigenesis.

Here, we studied the form-deprivation (FD) model of myopia in the mouse, primarily focusing on scleral end points described below. We hypothesized that myopigenesis in the mouse progresses via a retinoscleral signaling cascade similar to other mammals, and thus, that the sclera would be measurably more extensible in myopic eyes. Additionally, we hypothesized that sGAG content, shown to be rapidly altered in other species in response to myopigenic visual cues, would be decreased. Finally, we measured ocular atRA content to determine if increased retinal or choroidal atRA concentrations coincided with myopigenesis, as has been observed in other mammalian models.

## Materials and Methods

### Animals

Male C57BL/6J mice (Jackson Laboratory, Bar Harbor, ME, USA) were housed at the Atlanta Veterans Affairs Healthcare System in normal lighting conditions (12:12-hour cycles, 20-200 lux) with access to mouse chow and water ad libitum. All procedures were performed in accordance with the Association for Research in Vision and Ophthalmology (ARVO) Statement for the Use of Animals in Ophthalmic and Vision Research and approved by the relevant Institutional Animal Care and Use Committee.

### In Vivo Protocols

#### Ocular Measurements

At baseline and on the day of euthanization, animals were prepared for in vivo measurements.^37^^,^^38^ Eyes were dilated with 1% tropicamide and animals were anesthetized (ketamine = 80 mg/kg and xylazine = 16 mg/kg). Refractive error (RE; mouse automated infrared photorefractor), corneal curvature (CC; infrared photokeratometry), and ocular biometry (spectral domain optical coherence tomography [SD-OCT]; Envisu R4300, Leica Microsystems, Wetzlar, Germany) were then sequentially measured, with the entire process taking approximately 20 minutes.^35^^,^^39^ OCT volumes were registered to correct for motion artifacts, and tissue interfaces were manually marked, permitting the calculation of central corneal thickness (CCT), anterior chamber depth (ACD), lens thickness (LT), vitreous chamber depth (VCD), retinal thickness (RT), and axial length (AL; corneal surface to retinal pigment epithelium [RPE]). To convert optical path lengths from SD-OCT to physical distances, an average refractive index of 1.39 was assumed, resulting in 4.1 µm axial resolution in tissue. In vivo measurements were excluded when significant corneal/lens opacities were observed.

#### Induction of Form-Deprivation Myopia

After the baseline in vivo ocular measurements were made at postnatal day 28 (p28), FD myopia was induced by head mounting diffuser lenses unilaterally OD, leaving OS as an untreated contralateral control.^37^ Lens compliance was checked at least once daily, and no mice were eliminated from the study based on lack of lens compliance. Lenses were cleaned weekly under dim lighting and maintained for either 1 week (FD-1) or 3 weeks (FD-3).

### Ex Vivo Protocols

Following the final in vivo measurement, animals were euthanized, and eyes were enucleated and processed on the same day. Specifically, depending on the animal's assigned ex vivo end point, the eyes were processed in one of three ways:

1. Biomechanical characterization using unconfined compression (UCT);
2. Frozen for later quantification of total scleral sGAG content by 1,9-dimethylmethylene blue assay (DMMB) or for later atRA measurements using liquid chromatography-tandem mass spectrometry (LC-MS/MS); or
3. Fixed for immunohistochemistry (IHC).

**Figure 1.**
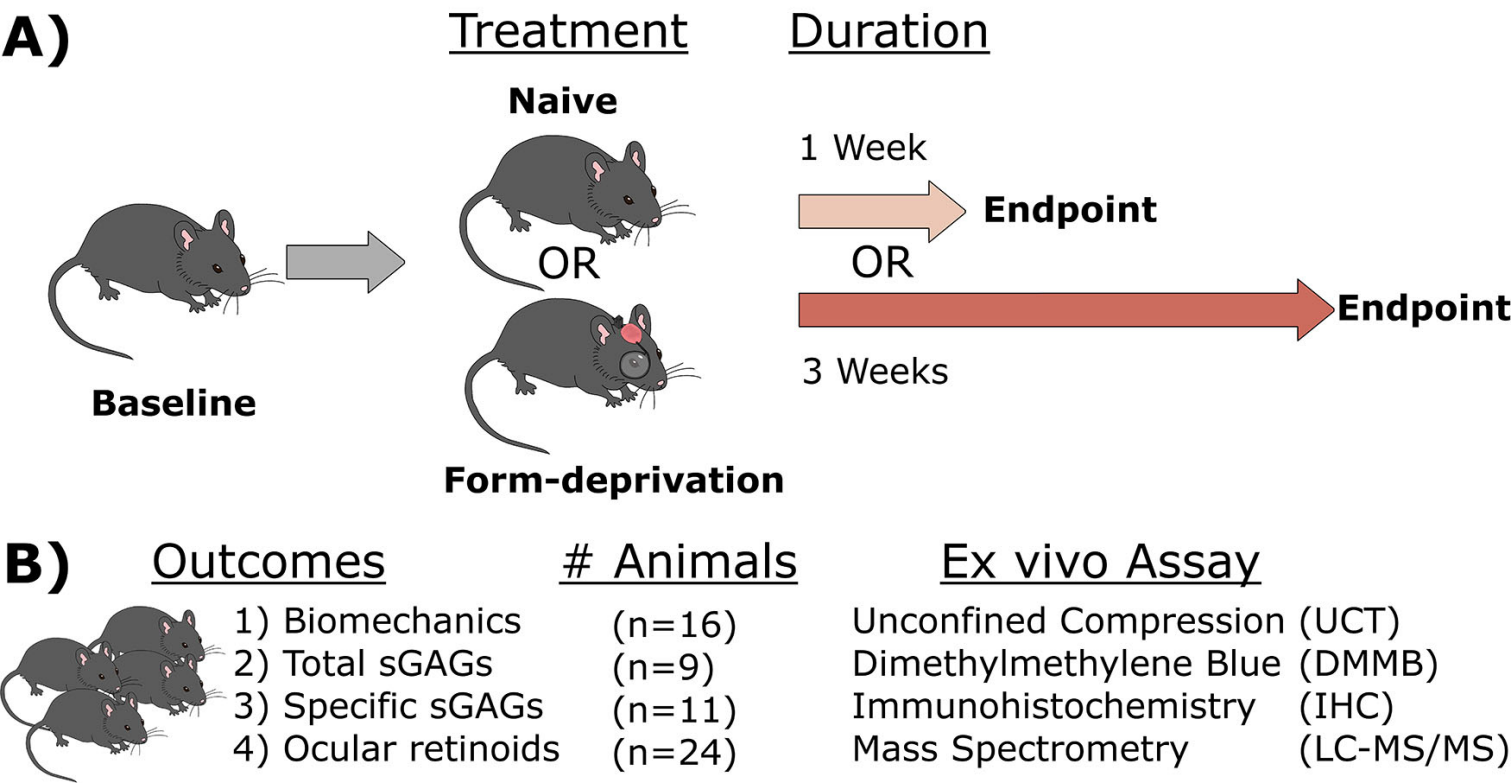
Experimental study design. (**A**) In vivo measurements were made at baseline, after which animals were either assigned to receive monocular form deprivation (FD) or remain naïve controls (naïve). Treatments were maintained for either 1 or 3 weeks, after which in vivo measurements were again made at the end point and prior to euthanization. (**B**) Ex vivo outcome measures were studied using different cohorts of animals.

Both eyes from each animal were used for the same ex vivo end points to permit pairwise comparisons within each animal and the calculation of a “shift” or interocular difference (IOD; treated minus contralateral eye). For some end points, untreated, naïve animals were also tested. The investigators were blinded in the DMMB and LC-MS/MS assays, and partially blind in IHC (samples were coded by the investigator and only decoded after imaging and masking was performed). In total, 60 animals were studied between the 4 different ex vivo end points (Fig. 1).

#### Unconfined Compression Testing

Eyes used for quantifying scleral biomechanics were enucleated and stored in phosphate-buffered saline (PBS) at 4°C until use the same day. As previously described,^40^ eyes were first dissected under PBS to remove orbital muscle, fat, and conjunctiva. Globes were divided at the limbus, and the intraocular tissues were removed to create a scleral shell. Four cuts were made to flatten the sclera and create four regions from which samples could be taken. Then, 1 mm diameter cylindrical samples were taken from the posterior region of the scleral shell using a biopsy punch, approximately 0.5 mm from the optic nerve. Samples were taken randomly from the four regions, and when multiple samples were tested from the same eye, the results were averaged. In support of this approach, in the current study and our previous study^40^ detailing the methodology, we have seen no effect of region on measured mechanical properties. Samples were coated in graphite powder and submerged in a 37°C 1 times PBS fluid bath for biomechanical testing.

UCT was performed on the cylindrical samples using a cantilever-based compression testing apparatus (Microsquisher; CellScale, Waterloo, Ontario, Canada). Samples were placed on the platens and a 500 µN (636 Pa engineering stress) tare load was applied to flatten the samples and keep them in place. After equilibration, the thickness of the sample was measured via the calibrated camera (resolution = 0.6155 µm/pixel). This was used as the reference configuration for stress/strain calculations. Then, an incremental 3 step stress relaxation compression protocol was performed, applying a total of 15% compressive strain over 3 steps, with each step applying and maintaining an additional 5% compressive strain until stress relaxed and equilibrated (the average relaxation time = step 1: 12 minutes, step 2: 22 minutes, and step 3: 25 minutes). The time taken to relax increases with each incremental strain step due to the viscoelastic effects being a result of fluid exudation, and the tissue hydraulic conductivity is dependent on the state of compression (i.e. increased compression causes pores to close and resistance to fluid flow to increase). The biphasic conewise linear elastic material model was independently fit to each step of the compression experiment, permitting the calculation of an “aggregate” tensile modulus (intrinsic material property reflecting resistance to tensile deformation once internal fluid flow has ceased; “tensile stiffness”) and hydraulic conductivity (tissue permeability to water; “permeability”) at each of the three steps^41^^,^^40^(Supplementary Fig. S1).

The IOD of each material property was calculated at each step and averaged to obtain the mean IOD of each sample. However, in some cases, applied compressive strain deviated up to approximately 1.5 percentage points from the target strain (see Supplementary Fig. S1). Due to the strong dependence of the properties on the state of sample compression, we included this deviation as a factor in our statistical model (generalized linear models). IODs are presented as estimated marginal means of the fitted statistical model in which the strain deviations are corrected.

#### Dimethylmethylene Blue Assay

To quantify total scleral sGAG content, eyes were enucleated and stored in PBS at 4°C until dissection the same day. The sclera was isolated from the rest of the eye while submerged in PBS, carefully removing the RPE and choroid by scraping with the tips of angled forceps. Isolated sclerae were then dehydrated in a vacuum desiccator overnight and weighed three times on an analytical balance (XSR205DU; Mettler Toledo, Columbus, OH, USA), with the average being taken as the dry mass of the sclera. These procedures led to good interocular agreement in tissue masses in a preliminary group of naïve, untreated animals (*n* = 8 pairs of eyes, concordance correlation coefficient ρ_c_ = 0.94; Supplementary Fig. S2). The isolated sclerae were frozen on dry ice in Eppendorf tubes and stored at −80°C until use.

Total sGAG content was measured via the metachromatic DMMB assay. Samples were first thawed and digested in 200 µL papain (Sigma P3125, 2% v/v) in buffer (0.1 M sodium acetate, 0.01 M cysteine hydrochloride, 0.05M EDTA, and pH 6.0) at 60°C for 1 to 2 days, or until completely digested. Samples were spun down every 4 to 6 hours to keep them immersed. Then, 200 µL of the DMMB dye mixture (8 mg 1,9 dimethylmethylene blue in 2.4 mL ethanol, 1 g sodium formate, and 1 mL formic acid, diluted to 500 mL with ddH2O) was added to 30 µL of digested sample in a 96 well plate and absorbance was read at 525 nm, the accepted preferred wavelength for measuring sGAG content with this assay.^42^ Concentration was determined by comparing absorbances to a standard curve created from shark cartilage chondroitin sulfate (Sigma C4384). DNA was also measured in each sample via the picogreen assay. Then, 100 µL of the picogreen working reagent (50 µL PicoGreen Reagent, 0.5 mL 20 times TE buffer, and 9.45 mL ddH20) was added to 5 µL of digested sample, and absorbance was read using 480 nm excitation and 520 nm emission and compared to a standard curve created using Lambda DNA stock (Cat #: SD0011; Thermo Fisher Scientific, Waltham, MA, USA). Both assays were performed in triplicate, with the average of the three taken as the final value.

To account for variability in the amount of isolated sclera collected per eye, scleral sGAG content is reported as a mass fraction (µg sGAG/mg sclera) and a mass ratio (µg sGAG/µg DNA).

#### Immunohistochemistry

Immediately following euthanization, eyes were immersion fixed in buffered zinc formalin (Z-Fix; Anatech LTD, Battle Creek, MI, USA) for approximately 1 hour. Eyes were cryoprotected in 30% sucrose solution overnight, embedded in optimal cutting temperature medium, and rapidly frozen at −60°C on the Peltier cooling element of a cryostat (CM1850; Leica Microsystems, Wetzlar, Germany). Then, 10 µm sagittal sections were taken through the optic nerve head and stored at −20°C.

For immunostaining, we used a well characterized monoclonal primary antibody (2B6) specific to chondroitinase-generated neoepitopes on both chondroitin-4-sulfate (C-4-S) and dermatan sulfate (DS) (Clone 2B6, Cat: 1042009, Lot: S1605002; MD Biosciences, Oakdale, MN, USA). C-4-S and DS make up the majority of the sGAG content in the sclera.^10^ Additionally, they tend to associate with two separate classes of proteoglycans; DS tends to be found predominately in small leucine-rich proteoglycans that associate with collagen (e.g. decorin and biglycan), whereas the majority of C-4-S tends to be found in larger, aggregating proteoglycans (e.g. aggrecan).^6^^,^^43^

To separately label C-4-S and DS, tissue sections were deglycosylated using either chondroitinase AC (ChAC), which specifically deglycosylates chondroitin A (C-4-S) and chondroitin C (C-6-S); or chondroitinase B (ChB), which targets DS (both ChB and ChAC from Flavobacterium heparinum; Sigma-Aldrich, St. Louis, MO, USA). Thus, tissue sections treated with ChAC and stained with 2B6 allow visualization of only the “stub” neoepitopes of C-4-S, and those treated with ChB and stained with 2B6 only allow visualization of DS (see the Table).

**Table.** Enzymatic Deglycosylations and Immunostaining Outcomes. Chondroitinase Enzymes Deglycosylate and Expose Neoepitopes on Specific sGAGs. Combining the Different Treatments With Antibodies (2B6 - Anti-4 Sulfation Stub Monoclonal Antibody) Permit Labeling of Specific sGAGs. sGAG, Sulfated Glycosaminoglycan; ChAC, Chondroitinase AC; ChB, Chondroitinase B; C-4-S, Chondroitin-4-Sulfate; C-6-S, Chondroitin-6-Sulfate; DS, Dermatan Sulfate

*Deglycosylation*		*Immunostaining*		
Enzyme	Exposed Neoepitope(s)	Antibody	Recognized Neoepitopes	Labeled GAG
ChAC	C-4-S	2B6	C-4-S	**C-4-S**
	C-6-S		DS	
ChB	DS	2B6	C-4-S	**DS**
			DS	

To prepare tissue for immunostaining, slides were first washed with tris-buffered saline with 0.1% Triton X-100 (TBS-T) to remove excess embedding medium. After enzymatic treatment (ChB, ChAC, or buffer-only; 0.5 U/mL, 3 hours at 24°C, 8.0 pH) sections were permeabilized (0.5% Triton X-100 in TBS, 10 minutes at 24°C) and then incubated with blocking buffer (10% normal goat serum and 1% bovine serum albumin in TBS-T, 45 minutes at 24°C). Sections were then incubated with mouse monoclonal antibody 2B6 in a humidity-controlled environment (1:100, 18 hours at 4°C). The following day, sections were washed 3 times in TBS-T and incubated with Alexa Fluor-647 labeled goat anti-mouse secondary antibodies in blocking buffer (1:500, 45 minutes at 24°C in darkness; Cat # A-21244; Invitrogen). Tissue was washed, treated with DAPI (NucBlue Fixed Cell Stain ReadyProbes DAPI, Cat # R37606; Invitrogen), washed, and finally coverslipped with antifade medium (Prolong Diamond Antifade Medium, Cat # P36961; Invitrogen). All slides were stained in parallel using the same batch of working reagents.

Stained samples were imaged on a confocal microscope (Nikon A1R; Nikon, Tokyo, Japan) using an apochromatic 20× objective lens. Three images (600 × 600 µm per field of view, 12 bits per channel) were taken per section – one centered on the optic nerve head, and one each superior and inferior to the first. Three sections were imaged per slide (ChB-treated, ChAC-treated, and buffer-only control). Microscope settings were kept constant across all samples and imaging sessions.

Images were stitched, masked, thresholded, and quantified via custom semiautomated macros using built-in analysis functions in FIJI.^44^ The sclera was manually masked from other tissues. To quantify relative GAG content, average pixel intensities were measured within the masked regions and normalized to each slide's buffer-only control.

#### Liquid Chromatography Tandem Mass Spectrometry

Eyes used to quantify retinoids were enucleated and dissected in PBS under dim red light to limit RA isomerization.^45^^,^^46^ The extraocular tissues, optic nerve head, anterior chamber, and lens were removed from the eyes. The remaining posterior cup was divided into two groups by carefully isolating the retina. The remaining RPE, choroid, and sclera (R/C/S) were analyzed together due to the small quantities of tissue (mean ± standard deviation sample mass; retina = 14.6 ± 4.8 mg, and R/C/S = 10.8 ± 3.3 mg). Tissues were blotted, placed into light proof Eppendorf tubes on dry ice, and stored at −80°C.

Samples were shipped from Atlanta, Georgia, to Baltimore, Maryland, on dry ice for LC-MS/MS characterization of retinoids using a previously detailed and rigorously characterized methodology, developed specifically for measuring endogenous RA isomers in limited tissue samples from mice.^45^^–^^49^ In brief, samples were combined, and the wet weights of the grouped samples were measured. Tissue was then homogenized in a ground glass homogenizer in 0.9% NaCl (normal saline) and extracted using a 2-step acid-base extraction previously described in detail using 4,4-dimethyl-RA as an internal standard for RA and retinyl acetate as an internal standard for retinol and total retinyl esters.^46^ Levels of RA were quantified using liquid chromatography-multistage-tandem mass spectrometry, which is an LC-MS/MS method utilizing two distinct fragmentation events for enhanced selectivity using a Prominence UFLC XR liquid chromatography system (Shimadzu, Columbia, MD, USA) coupled to a 6500+ QTRAP hybrid triple quadrupole mass spectrometer (AB Sciex, Framingham, MA, USA) using atmospheric chemical ionization (APCI) operated in positive ion mode as previously described.^49^ Levels of retinol and total retinyl esters were quantified via HPLC-UV, as previously described.^46^^,^^48^ From these procedures, atRA, retinol (ROL), and total retinyl esters (tRE) were quantified and reported. Individuals carrying out the LC-MS/MS measurements were blinded to the identities of the samples.

### Statistical Analysis

Generalized linear mixed effects models were constructed for each outcome measure. Fitting and contrasts were done using the “lme4” and “emmeans” packages in R (version 4.1.1).^50^^,^^51^ When multiple contrasts were run, the *P* values were adjusted via the multivariate t method.^52^ Significance of fixed effects was calculated by performing likelihood ratio tests between the full model and null models, each lacking one of the fixed effect terms. Random effects were used to account for correlated samples (e.g. 2 eyes from the same animal or multiple measurements on the same eyes). Outcomes were assigned a Gamma error distribution with a log link based on priors (outcomes that are strictly positive and/or ratios) and based on analysis of model residuals (Supplementary Table S1). Significant alpha threshold was taken to be 0.05, and all results are presented as mean ± standard deviation.

## Results

### Form Deprivation Induces Myopic Refractive Errors in Mice

**Figure 2.**
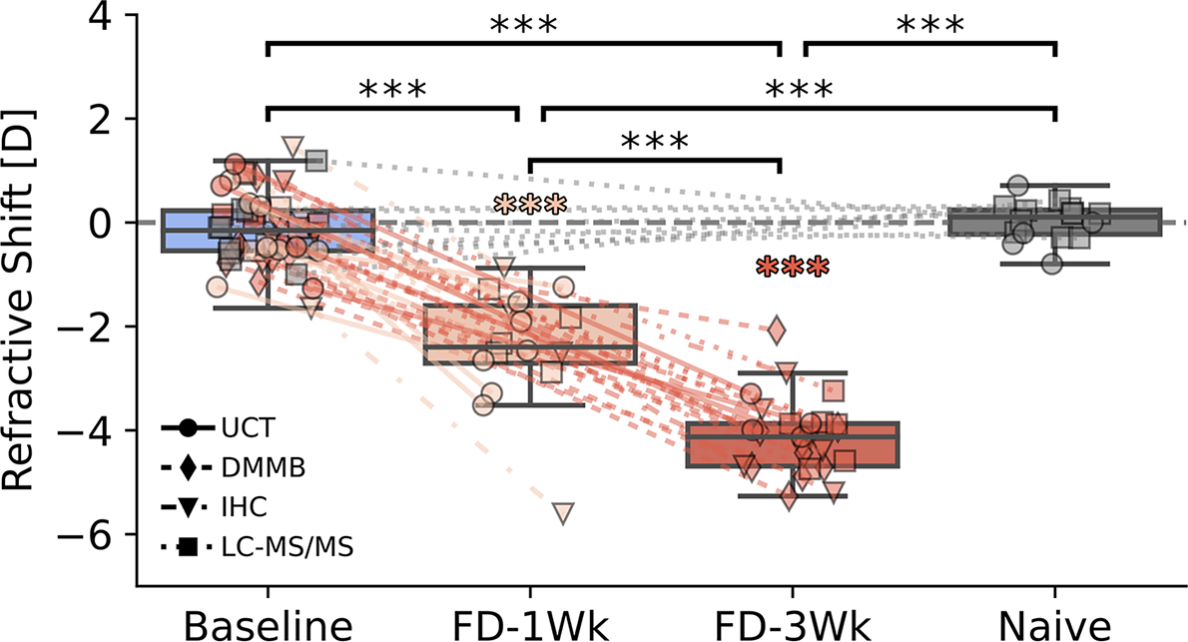
Form deprivation caused time-dependent myopigenesis in mice quantified by refractive error. Naïve animals and all animals at baseline displayed good interocular agreement in refractive error (treated – contralateral close to 0). Form deprivation caused increasingly more myopia over time relative to the contralateral eye. Different symbols represent the different outcome measures studied in each animal. Brackets denote difference between groups; filled asterisks denote an interocular difference in that group. The box contains the second and third quartiles of data, with the line indicating the median. The whiskers show the first and fourth quartiles. UCT, unconfined compression test; DMMB, dimethylmethylene blue; IHC, immunohistochemistry; LC-MS/MS, liquid chromatography tandem mass spectrometry. *: *P* < 0.05, **: *P* < 0.01, ***: *P* < 0.001.

At baseline and in the naïve animals, refractive errors displayed good interocular agreement (i.e. refractive shifts [IOD RE] were not significantly different from 0 [baseline = −0.13 ± 0.67 D and naïve = 0.02 ± 0.37 D). Form-deprivation, initiated at 4 weeks of age, led to myopia relative to the contralateral eye by 1 week (−2.35 ± 1.11 D, *P* < 0.001) and worsened by 3 weeks (−4.07 ± 0.72 D, *P* < 0.001; Fig. 2). Despite myopic refractive errors, no biometry measurements were significantly altered with myopia relative to their contralateral controls, including AL (Supplementary Table S2).

### Scleral Stiffness and Permeability Are Both Altered During Myopigenesis

**Figure 3.**
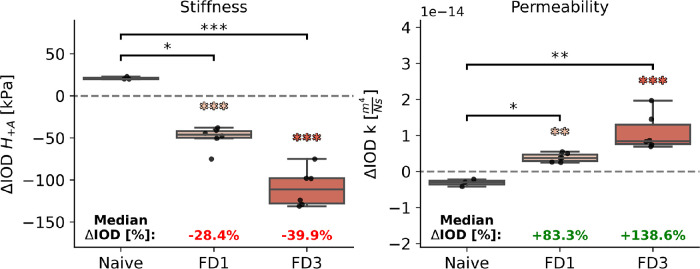
Form deprivation altered the biomechanics of the sclera, decreasing the tensile stiffness and increasing permeability as myopia develops relative to the contralateral eye. In other words, the interocular differences in scleral aggregate tensile modulus (*left*) and hydraulic conductivity (i.e. permeability) (*right*) differed from 0 due to form deprivation. Interocular differences were calculated at each step and the differences were averaged. Values plotted are the estimated marginal means derived from the fitted statistical model. The *gray dashed line* shows the 0 point (no interocular difference). *Filled asterisks* indicate a significant difference from 0. The *box* contains the second and third quartiles of data, with the *line* indicating the median. The whiskers show the first and fourth quartiles. *Brackets* indicate differences between groups. FD1, 1 week form deprivation; FD3, 3 weeks form deprivation; IOD, interocular difference; H_+A_, aggregate tensile modulus (“stiffness”); k, hydraulic conductivity (“permeability”). *: *P* < 0.05, **: *P* < 0.01, ***: *P* < 0.001.

In naïve animals, the measured tensile stiffness and permeability displayed good interocular agreement, with only minor and insignificant differences occurring. The sclerae from form-deprived eyes were less stiff (FD versus contra; 1 week FD = −29.2%, *P* < 0.001, and 3 weeks FD = −39.9%, *P* < 0.001) and more permeable (FD versus contra; 1 week FD = +80.1%, *P* = 0.0025, and 3 weeks FD = +131.4%, *P* < 0.001) than the contralateral eyes (Fig. 3). The interocular differences in both FD groups were significantly different from those measured in the naïve animals (naïve versus FD-1: stiffness, *P* = 0.02; permeability: *P* = 0.04; and naïve versus FD-3: stiffness, *P* < 0.001; permeability: *P* < 0.001). Scleral biomechanical properties did not differ between contralateral eyes of either FD group or between contralateral eyes and naïve eyes (see Supplementary Fig. S1, Supplementary Table S3).

### sGAG Content in the Posterior Sclera Is Decreased With Myopia Development

**Figure 4.**
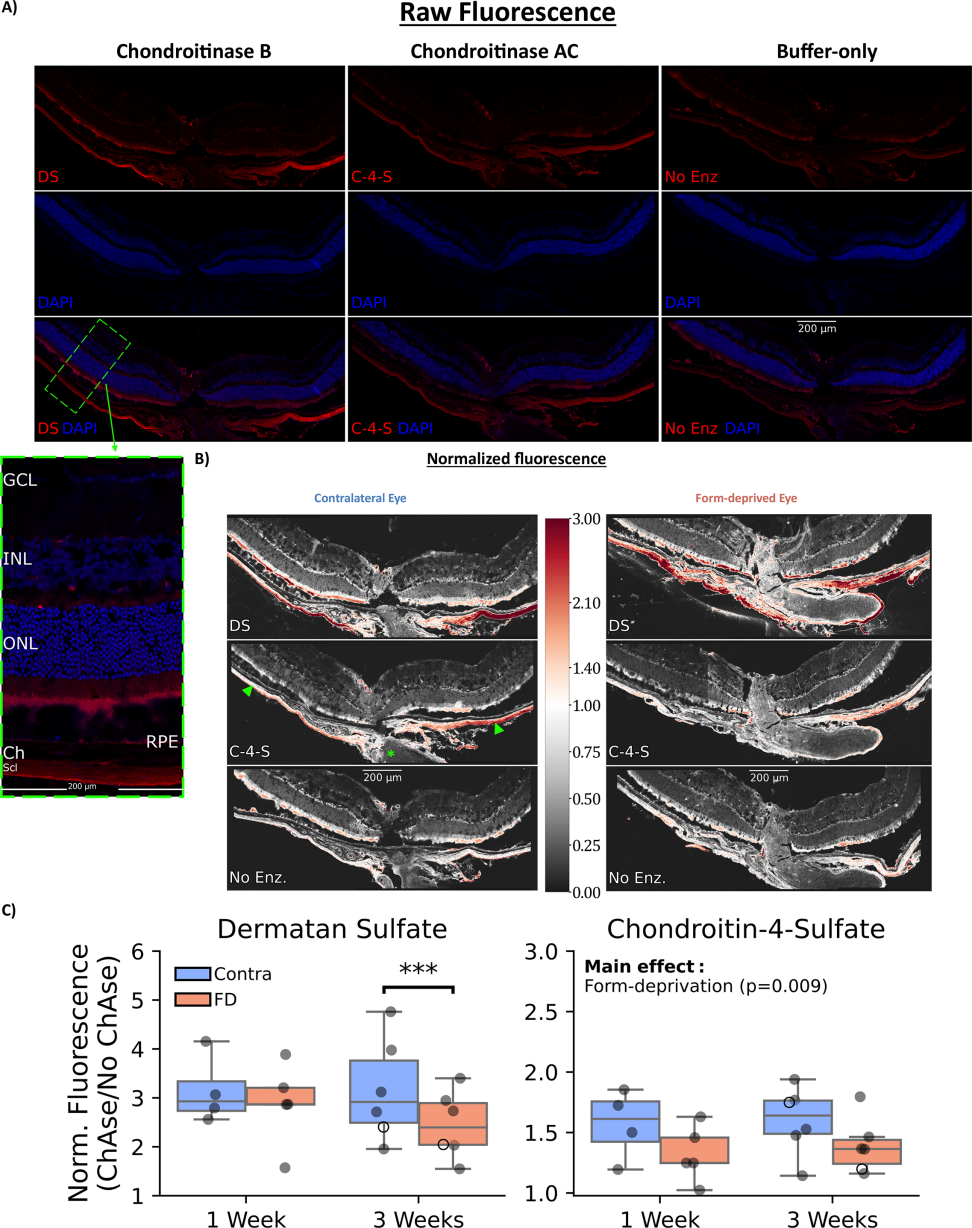
Effect of form deprivation on immunostaining of specific scleral sGAGs. (**A**) Representative immunostaining of the posterior eye. Without chondroitinase treatment, staining was minimal (*right*). Deglycosylation using ChAC (*middle*) or ChB (*left*) increased the amount of labeling, primarily in the sclera. The *green dashed rectangle* shows an annotated/magnified 200 µm wide region and is representative of the distance from the optic nerve at which staining was quantified. (**B**) Representative normalized fluorescence of the same sample in **A** presented alongside the same animal's form-deprived eye. Fluorescence was normalized to the average intensity in the sclera of the “buffer-only” treatment to account for inter-sample differences in nonspecific staining and autofluorescence. *Red* coloring indicates increased staining, *white* indicates no change, and *gray* coloring indicates decreased staining relative to the sclera of the “buffer-only” treatment. *Arrowheads* point to the sclera and the *asterisk* labels the optic nerve. (**C**) Quantification of the normalized fluorescence. DS content was significantly decreased in the sclera only after 3 weeks of form deprivation. In contrast, C-4-S content was significantly decreased in form-deprived eyes independent of the treatment duration (main effect: treatment, *P* < 0.001). The *box* contains the second and third quartiles of data, with the *line* indicating the median. The whiskers show the first and fourth quartiles. Open markers show the spatially averaged values of the representative samples in **B**. *: *P* < 0.05, **: *P* < 0.01, ***: *P* < 0.001.

Total scleral sGAG content as quantified by the DMMB assay was not measurably altered after 3 weeks of FD (Supplementary Table S4). However, using semiquantitative immunohistological methods limited to the posterior sclera to observe the primary sGAGs, DS, and C-4-S, myopic sclerae tended to have decreased sGAG content (Fig. 4). DS content was dependent on the duration of FD (interaction effect: animal_treatment x eye, *P* = 0.017). DS was not changed in the FD eye relative to the contralateral eye after 1 week of treatment (*P* = 0.871) but decreased after 3 weeks (*P* < 0.001; Supplementary Table S5). A different trend was observed with C-4-S, which was decreased by a similar degree after both 1 and 3 weeks of FD (main effect: eye, *P* < 0.001; see Supplementary Table S5).

### Form Deprivation Alters Retinoids in Ocular Tissues

**Figure 5.**
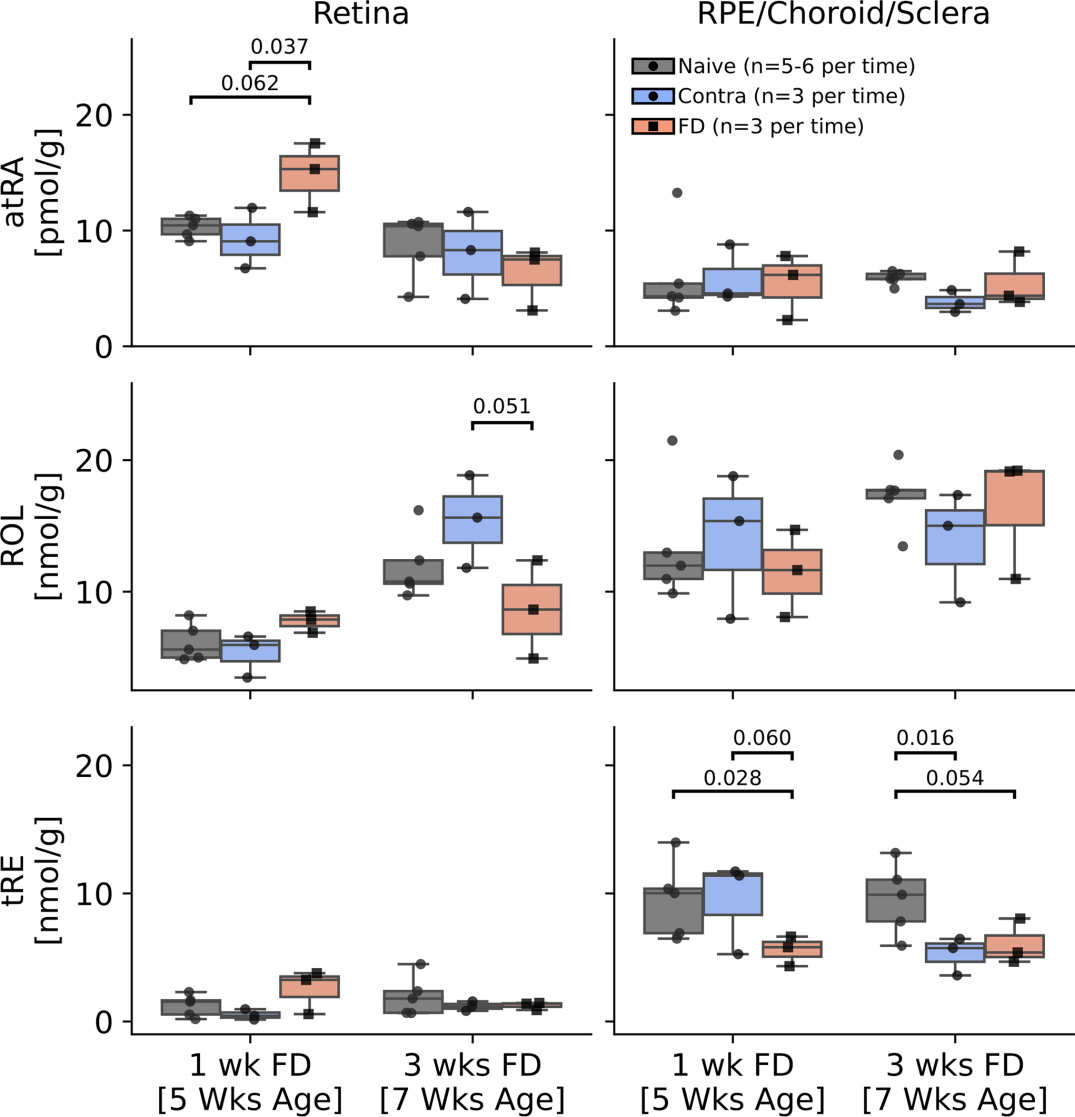
Retinoid concentrations in ocular tissues were altered with form deprivation. atRA increased in the retina of form-deprived eyes at 1 week. tRE decreased in the sclera of form-deprived animals - unilaterally after 1 week and bilaterally after 3 weeks. Each plotted point is one measurement made on grouped tissue from two eyes. Values above brackets are the adjusted *P* values of the simple effect contrasts of eye treatment (naïve, contra, or FD). The *box* contains the second and third quartiles of data, with the *line* indicating the median. The whiskers show the first and fourth quartiles. FD, form deprivation; Contra, contralateral; atRA, all-trans retinoic acid; tRE, total retinyl esters; ROL, retinol.

Retinoids (atRA, tRE, and ROL) were each quantified in the retina and combined R/C/S (Fig. 5). The bioactive retinoid, atRA, was significantly increased in the retina of eyes exposed to FD for 1 week compared to contralateral controls (*P* = 0.037), which returned to normal levels by 3 weeks. The concentration of atRA in the combined R/C/S was not altered with treatment. tRE in the R/C/S was decreased at 1 week of FD relative to naïve animals (naïve versus FD, *P* = 0.028), and a decrease was observed in the contralateral eyes at 3 weeks (naïve versus contra, *P* = 0.016).

## Discussion

The present study has established that the sclera of the mouse is significantly altered during visually mediated myopigenesis. A myopigenic visual cue (FD) presented to the retina leads to both myopic refractive errors and altered scleral biomechanics. The myopic mouse sclera is more extensible, consistent with studies in other species using disparate mechanical testing methods and measures of stiffness,^8^^,^^9^^,^^12^^,^^13^ and more permeable, a finding not previously reported. In our efforts to quantify degradation of sGAGs, results from semiquantitative IHC suggests that the two major scleral sGAGs, C-4-S and DS, are decreased in the posterior sclera. Interestingly, DS was only reduced after 3 weeks of FD, whereas significant myopia, altered biomechanics, and reduced C-4-S were found even at 1 week of FD, suggesting that C-4-S, but not DS, may be involved in the early process of axial elongation. Finally, we present an exploratory overview of retinoid concentrations in ocular tissues of mice, which appear to vary throughout the posterior eye wall in response to FD. These findings together, despite having not measured an increase in axial length, support the hypothesis that the mouse develops axial myopia via a retinoscleral signaling pathway, with largely conserved scleral endpoints to those reported in other mammalian species.

### The Mouse Sclera Becomes More Extensible and Permeable During Myopigenesis

In contrast to the large variability of axial length (standard deviation of interocular difference approximately 25 µm, or an estimated approximately 5 D in the mouse), the effects of FD on the scleral material properties are large and consistent. One week of FD resulted in approximately 2 D of myopia and corresponded with a 30% increase in the median extensibility of the myopic sclera, relative to the contralateral eye. By 3 weeks, the amount of myopia nearly doubled to approximately 4 D and myopic sclerae were approximately 40% more extensible. Without any adjustments from the statistical model, 1 week of FD caused 5 to 6 animals to have more extensible myopic sclerae, increasing to 6 of 6 animals by 3 weeks of FD. The trend of a more extensible sclera in myopic eyes is consistent with previous findings of tensile stiffness changes in the chicken and tree shrew sclera, measured using a variety of approaches.^12^^,^^13^ Although it is still not clear how exactly stiffness relates to axial length or what specific remodeling processes influence stiffness, these findings support the idea that the elongation is not driven by strictly elastic deformations of the corneoscleral shell.

The permeability of the mouse sclera was also altered in response to FD, a finding not previously reported in any species. The tissue permeability of hydrated tissues accounts for a significant amount of viscoelasticity (time-dependent responses to loading) under certain modes of deformation,^53^^,^^54^ and the eye is constantly experiencing pulsatile, time-varying loading via the intraocular pressure and normal eye movements.^55^ Thus, whereas it is feasible that scleral permeability could have important implications for scleral deformations, it is not obvious what role, if any, permeability may play in the proper functioning of the sclera and regulation of eye size. We note though that permeability appeared to track myopia better than tensile stiffness in this study; both myopic refractive errors and median permeability increased by about 70% between 1 and 3 weeks (−2.4 D to −4.1 D, +83% to +139% relative to the contralateral eye), whereas median tensile stiffness decreased by only approximately 40% (−28% to −40%).

Despite lacking a clear understanding of this functional relationship, measurements of the scleral permeability may serve as a useful proxy for characterizing scleral microstructure, specifically its sGAG content. Tissue permeability has been most extensively studied in articular cartilage, where permeability is directly tied to function (i.e. in the lubrication of joints).^56^ Theoretical and experimental results support a negative correlation between permeability and sGAG content^57^^,^^58^ (i.e. decreased sGAG content results in increased permeability). If a similar relationship is demonstrated in the sclera, the measured increase of permeability in this study would be consistent with the widely replicated finding that scleral sGAGs decrease during myopigenesis.^10^^,^^59^

### Specific Scleral Glycosaminoglycans Are Decreased With Different Time Courses

To study aspects of remodeling that occur in the mouse sclera during myopigenesis, we characterized sGAG content using two assays: DMMB (quantitative) and IHC (semiquantitative). Whereas total sGAG content was not found to change after 3 weeks of FD using the DMMB assay, the 2 most abundant sGAGs in the sclera, DS and C-4-S, appeared to be altered due to FD when studied using IHC.

We report that C-4-S content is decreased in the myopic mouse sclera, occurring by 1 week and persisting through 3 weeks. This decrease in response to FD agrees with a study by Moring et al., in which C-4-S was found to decrease at 4 days of lens defocus myopia in the tree shrew.^10^ C-4-S is primarily found on large, aggregating proteoglycans (e.g. aggrecan),^43^^,^^60^ and aggrecan production has been shown to be rapidly downregulated, whereas aggrecanases are upregulated in the sclera in response to myopigenic visual cues.^61^^–^^63^ Thus, the semiquantitative results of the IHC are consistent with other methods and species.

Additionally, we found that scleral DS was decreased, but only after 3 weeks of FD. DS has been shown to associate closely with, and influence, collagen, with DS concentrations being correlated to the amount of collagen.^10^^,^^64^^–^^69^ Thus, this decrease in DS may be indicative of longer-term decreases in type I collagen content, a conserved finding across species.^70^

The apparent contradictory results obtained from the DMMB and IHC assays may be reconciled in several ways. First, the two assays measured different regions/amounts of the sclera. It was not feasible to isolate and measure a specific scleral region for the DMMB assay due to the very limited amount of scleral tissue from each mouse eye (approximately 150 µg of total, dehydrated tissue). Thus, whereas total scleral samples were used in the DMMB assay, we limited the IHC to the posterior sclera, where changes in scleral remodeling during myopigenesis have been most commonly shown.^10^^,^^59^ Other possible explanations could include lack of sensitivity of the DMMB assay to relatively small changes in total GAG and/or variability in the processing of the small tissue samples.

### A Speculative Mechanism Linking Scleral sGAGs to Biomechanics

By incorporating results from the present study with those from studies of cartilage biomechanics and scleral remodeling in myopia, it is possible to construct a speculative but self-consistent mechanism linking scleral sGAGs to biomechanics. In cartilage, permeability affects fluid pressurization in the tissue, hydration, and the loading imposed on collagen fibrils,^70^ all of which may influence collagen crimp and/or recruitment.^71^^–^^74^ During myopigenesis, aggrecan and sGAGs appear to be the most rapidly altered component in the sclera,^10^ and other work shows evidence of collagen crimp being rapidly increased in the myopic sclera.^13^ We hypothesize that loss of sGAGs in the sclera is causal to the increased tissue permeability, subsequently decreasing fluid pressurization and loading of the collagen fibrils, resulting in increased crimping of collagen fibrils that translates to a decrease in the tissue-level tensile modulus. We emphasize that results from cartilage are not guaranteed to hold in the sclera, because the relative proportion of sGAGs/proteoglycans to other extracellular components in the sclera is different from cartilage by approximately an order of magnitude. However, sGAGs and collagen crimp have also been shown to be related in tendon, which is more comparable to the sclera in terms of sGAG content (approximately 10% in articular cartilage, approximately 2.5% in tendon, and approximately 1% in the sclera) and other anatomic features.^74^^,^^75^

A logical outcome of the above proposed mechanism is that the scleral tensile modulus and sGAGs content are positively correlated, where sGAG content causally influences tensile stiffness. Attempts to study this structure-function question have utilized ex vivo methods to degrade sGAGs and measure changes in biomechanics; however, no consensus has been reached, with studies showing that degrading sGAGs increases,^76^^,^^77^ decreases,^77^^,^^78^ or has no effect on^79^ the tensile stiffness of the sclera. However, these studies digested significantly more GAGs, in quantity and type, than are altered with myopigenesis. Short-term decreases in scleral GAGs in myopia appear to be restricted to those associated with the aggregating proteoglycans (hyaluronan and C-4-S) that typically reside between the collagen lamellae; the above-referenced studies nearly completely digested all scleral sGAGs, including DS, which are typically attached to small leucine-rich proteoglycans that associate closely with and regulate collagen fibrils.^64^^–^^69^ Additionally, the sclera was loaded under tension via different methods in these studies; perhaps sGAGs differentially contribute to the biomechanical response to a directly applied tensile load^79^ versus tension resulting from compression or inflation.^76^^–^^78^ Further study of the solid/fluid biphasic mechanics of the sclera (as studied here), along with extending this work to study the sclera as a triphasic material (solid/fluid/ions), are warranted to elucidate these relationships.

### Ocular Retinoids Are Altered During Myopigenesis

One possible mechanism by which a visual cue may be propagated to the sclera is via retinoids, including atRA, the bioactive form of retinoic acid. Previous studies have shown myopigenesis in mammals is accompanied by increased concentrations of atRA in the retina and choroid. Here, we measured retinal and R/C/S concentrations of atRA, as well as ROL and tRE, atRA precursors typically understood to be used for transport and storage.^80^ Our results indicate that all three retinoids were influenced by FD, with potentially interesting and unexpected temporal characteristics. Specifically, after 1 week, we observed increases in retinal atRA consistent with previous findings in guinea pigs, chickens, and marmosets.^30^^,^^33^^,^^81^ However, this difference was not seen after 3 weeks of FD. The data in this study suggest that retinoids in the retina and at least one of the RPE, choroid, and/or sclera are being influenced by visual cues; however, future work using a larger cohorts of animals will be necessary to draw stronger conclusions about the role of atRA in mouse myopigenesis.

### Technical Challenges of the Mouse Model of Myopia

Significant effort was spent in the present study aligning, positioning, and processing OCT images to reduce extrinsic variability in biometric measurements, yet we were unable to measure an overall effect of FD on axial length. This negative finding is common in the mouse,^35^^,^^36^ raising concerns over whether the mouse develops axial myopia consistent with other mammals.

It is possible that the mouse does not undergo axial myopigenesis; however, the axial elongation could also be falling below the threshold of detection. Schematic models of the mouse eye have suggested that 1 D of myopia only requires about 5 µm of elongation.^82^ The axial resolution of a typical OCT system (approximately 4 µm) thus corresponds to approximately 1 D of myopia. In practice, biological variability (e.g. in eye size, refractive index, cataracts, and astigmatism) and noise inherent to the measurements (e.g. breathing artifacts and acute corneal/lens opacities, intersession/interocular alignment variability, magnification variability in non-telecentric lenses, and manual segmentation errors) will further raise the threshold of detection above the resolution.

Whereas there are reports of axial elongation in the myopic mouse eye,^35^^,^^38^^,^^83^ it is difficult to weight the positive and negative findings in an unbiased manner. Experimental studies reporting positive findings are not consistent in the amount of axial elongation per diopter of myopia (ranging from 5–40 µm/D), thus generally also inconsistent with the 5 µm/D estimate from modeling studies.^36^ Lacking a replicable, consistent effect and/or additional positive findings causal to elongation (e.g. scleral remodeling/biomechanics), it is difficult to conclude whether these studies show true positive findings or type I errors, possibly due to bias either at the level of the researchers (e.g. “lack of blinding” bias) or literature (e.g. “positive result bias”).

## Conclusion

The present study clearly shows the sclera undergoes remodeling that results in significantly altered biomechanics, in a manner facilitating axial elongation. When compared to axial length as an outcome measure of myopia, scleral biomechanics show a greater effect size and consistency, readily detectable even with modest amounts of myopia (approximately 2 D). This suggests that, at least at present, scleral biomechanics may be a more reliable measure of axial myopigenesis than biometry in the mouse. It also supports the idea that negative axial elongation findings are type II errors arising from the small magnitudes of elongation. With the knowledge that retinoids are changing throughout the eye in a time dependent manner, future studies should focus on more thoroughly elucidating the spatial and temporal characteristics. Additionally, transgenic mice could help to better understand the causal impact of these retinoids and specific proteoglycans (aggrecan) on the mouse sclera.

## Supplementary Material

Supplement 1
