# Supplementary material for: Altered Structure and Function of Murine Sclera in Form-Deprivation Myopia
Source: Invest Ophthalmol Vis Sci. 2022 Dec 13;63(13):13. doi: 10.1167/iovs.63.13.13 (PMC9753793; doi:10.1167/iovs.63.13.13)
Supplement: Supplement 1 [file iovs-63-13-13_s001.pdf]

Supplementary Materials

Altered structure and function of murine sclera in form-deprivation myopia

Brown et al., IOVS 2022

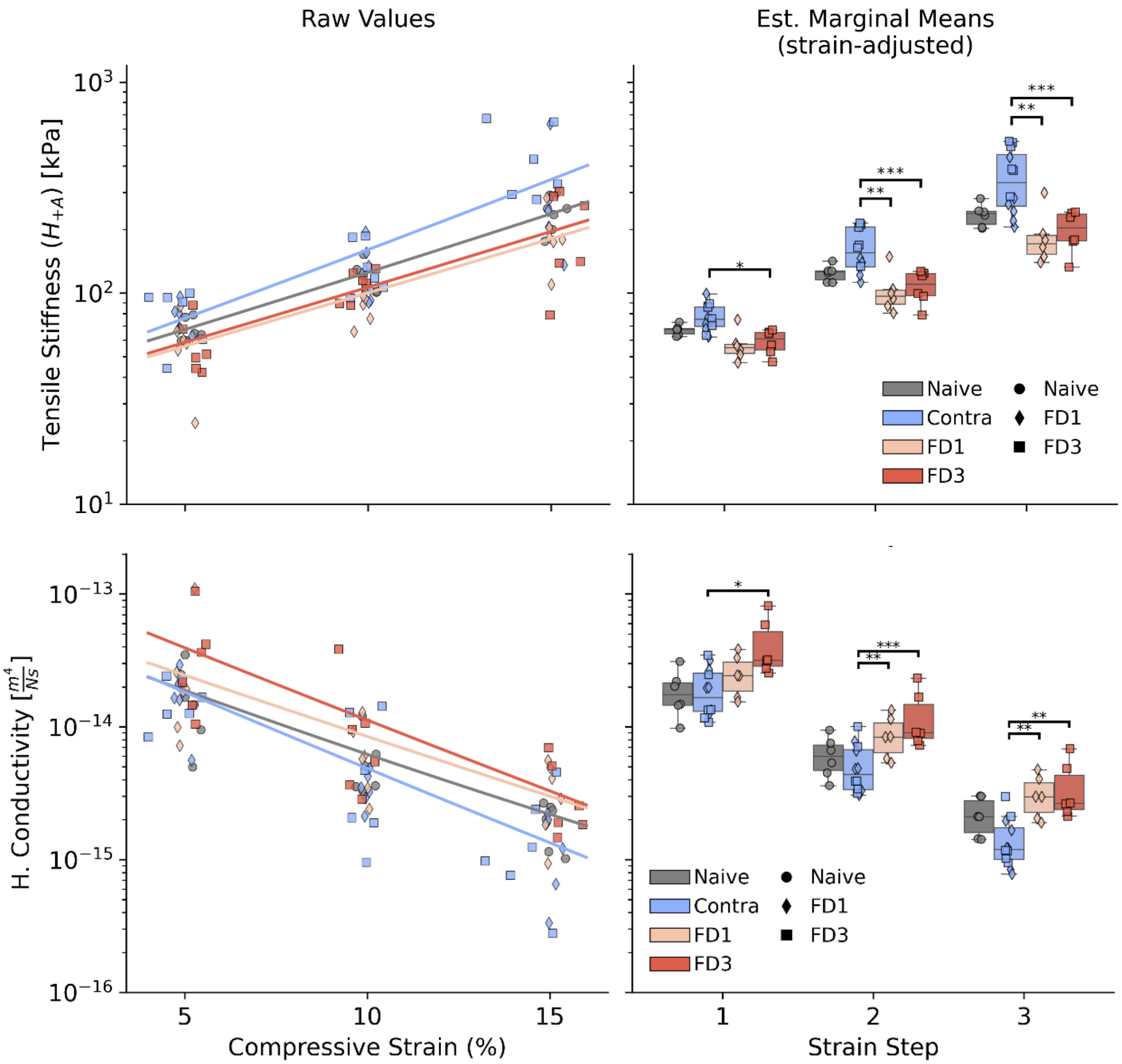

Figure S.1: The biomechanical material properties of the sclera were altered as a result of form deprivation. Specifically, aggregate tensile modulus (i.e., stiffness) (top) and hydraulic conductivity (i.e., permeability) (bottom) were both altered relative to the contralateral control eyes. Plots show the raw values obtained from fitting the poroelastic material model and the regressions obtained from the statistical model (left) and the estimated marginal means obtained from the statistical model (right), both plotted as a function of the compressive strain magnitude (strain steps). FD1: 1 Week form deprivation, FD3: 3 weeks form deprivation. *: p<0.05, **: p<0.01, ***: p<0.001.

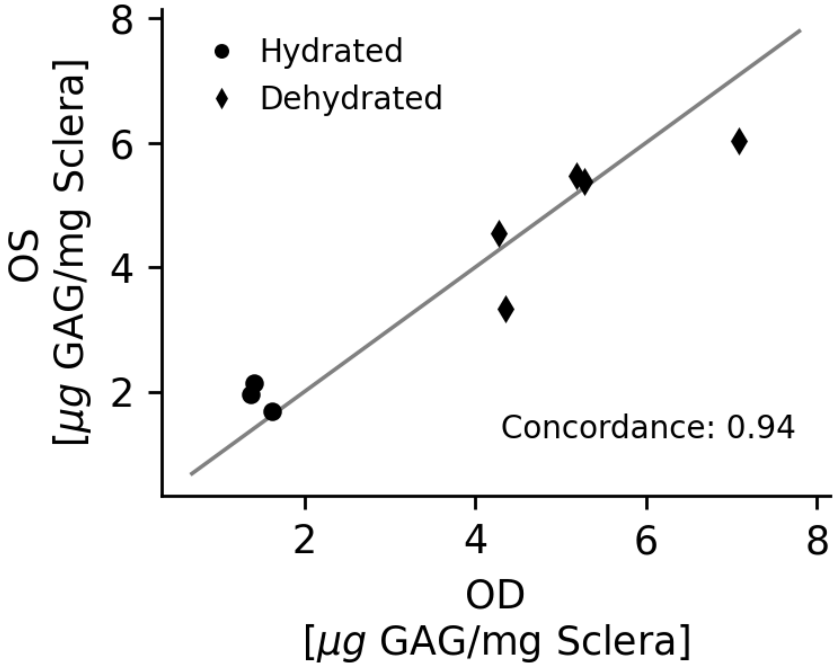

Figure S.2: Preliminary experiments demonstrate high concordance in GAG measurements made within control animals using the DMMB assay protocol. Each data point represents measurements of the scleral GAGs in the left (OS) and right (OD) eyes of an individual animal. Some samples were blotted (diamonds, hydrated) and others were dehydrated (circles, dehydrated). The DMMB assay measured differences in the two treatments and had high concordance across the entire range of concentrations (concordance = 0.94). The solid line represents the identity line (OD = OS). Perfect concordance (Concordance=1) would occur if every point fell on the identity line. DMMB: dimethylmethylene blue, GAG: glycosaminoglycans.

Table S.1: Summary of statistical models used to analyze outcome measures. Covariates identify continuous predictors set to 0 during analysis. Covariate days_deviation is the difference from timepoint in the in vivo measurements (4 weeks, 5 weeks, 7 weeks) or the mean age at the time of sacrifice. "*": full interaction operator indicating inclusion of all main effects and interactions, "1|animal_id": random intercept for each animal. UCT: unconfined compression test, DMMB: dimethylmethylene blue assay, PG: picogreen assay, IHC: immunohistochemistry, LC-MS/MS: Liquid chromatography tandem mass spectrometry, DVs: Dependent variables, RE: Refractive error, AL: Axial length, H_{+A}: Aggregate tensile modulus (tensile stiffness), k: permeability, GAG: glycosaminoglycan, atRA: all-trans retinoic acid, tRE: total retinyl esters, ROL: Retinol.

Outcomes	DVs	Fixed Effects	Covariates	Random Effects	Family	Link
**In Vivo**	RE, AL	~ age*animal_treatment*eye	days_deviation	1|animal_id	Gaussian	Identity
**UCT**	H_{+A}, k	~ animal_treatment*eye*strain	days_deviation	1|animal_id	Gamma	Log
**DMMB/PG**	GAG/mass, GAG/DNA	~ eye		1|animal_id	Gamma	Log
**IHC**	NormIntensity	~ animal_treatment*eye		1|animal_id	Gamma	Log
**LC-MS/MS**	atRA, tRE, ROL	~ age*eye_treatment*tissue			Gaussian	Identity

Table S.2: Effect of form deprivation on ocular biometry. Tabulated data are the mean ± standard deviation of interocular differences for each outcome. Bolded values are significantly different from 0 ($p<0.05$). RE: Refractive error, CC: Corneal curvature, CT: corneal thickness, ACD: Anterior chamber depth, LT: Lens thickness, VCD: Vitreous chamber depth, AL: Axial length

Group	RE [D]	CC [μm]	CT [μm]	ACD [μm]	LT [μm]	VCD [μm]	RT [μm]	AL [μm]
Baseline (53)	-0.13±0.67	-2.52±12.90	-1.31±6.86	-1.65±10.04	-3.50±13.90	2.83±16.12	0.06±10.73	-3.10±27.01
Naive (16)	0.02±0.37	-2.07±5.81	-1.56±2.58	1.61±6.97	-3.19±14.58	3.58±13.22	3.76±5.59	5.27±24.41
1 Week FD (17)	**-2.35±1.11**	-4.52±7.80	-1.28±4.48	-0.20±11.19	-4.40±12.40	3.48±16.62	-1.29±8.57	-0.89±27.29
3 Weeks FD (27)	**-4.07±0.72**	1.53±16.43	0.00±6.31	3.39±9.65	2.61±17.17	4.74±16.52	-2.96±10.15	9.87±25.05

Table S.3: Material properties obtained from 3 step unconfined compression testing of the mouse sclera. Tabulated values are the mean ± standard deviation of unadjusted, fitted material properties at each step, and averaged over the three-step protocol (~5%, 10%, 15% compressive strain). H_{+A}: Aggregate tensile modulus, k: permeability, FD1: 1 week FD, FD3: 3 weeks FD, Contra: Contralateral eye.

	$\boldsymbol{H_{+A}}$ [kPa]				$\boldsymbol{k}$ $[m^4/Pa \cdot s]$ (x10^{14})			
**Group (# eyes)**	**Step 1**	**Step 2**	**Step 3**	**Average**	**Step 1**	**Step 2**	**Step 3**	**Average**
**Naive (6)**	67.31±8.60	118.61±20.42	234.65±41.00	140.19±76.34	1.76±1.08	0.56±0.33	0.19±0.07	0.84±0.92
**Contra (12)**	78.75±17.82	132.21±38.85	371.23±182.99	194.06±166.53	1.72±0.68	0.48±0.43	0.15±0.12	0.78±0.82
**FD1 (6)**	58.10±20.21	92.72±23.70	189.32±55.90	113.38±66.85	3.18±3.89	0.66±0.39	0.34±0.18	1.39±2.49
**FD3 (6)**	57.18±17.55	109.83±17.86	201.74±93.63	122.92±80.88	3.84±3.49	1.18±1.35	0.33±0.22	1.78±2.55

Table S.4: Effect of form deprivation on total scleral sulfated GAG content as measured by the DMMB assay. Total sulfated GAG content was not significantly altered after 3 weeks of form deprivation when compared to contralateral control eyes ($p>0.05$ for all). GAG: glycosaminoglycans, DMMB: dimethylmethylene blue assay.

**Group**	**Scleral mass (dry)** [µg]	**Total GAG** [µg]	**GAG Conc.** [µg/mg]	**GAG/DNA** [µg/µg]
Contralateral (n=9)	161.78 ± 16.85	1.04 ± 0.10	6.54 ± 1.21	8.50 ± 0.94
Form-deprived (n=9)	167.78 ± 16.01	1.16 ± 0.17	7.01 ± 1.40	9.30 ± 1.72
Interocular Difference (n=9)	6.00 ± 26.05	0.12 ± 0.21	0.48 ± 2.33	0.80 ± 1.95

Table S.5: Effect of form deprivation on scleral sGAGs as measured by immunostaining of chondroitinase-treated samples. Chondroitin-4-sulfate content was significantly decreased in form-deprived eyes, independent of the duration of form deprivation treatment. Dermatan sulfate content was only significantly decreased after 3 weeks of form deprivation. Tabulated quantities are mean ± standard deviation. Groups are presented as Treatment-Duration (# eyes). Durations (1 and 3) are weeks of form deprivation treatment. Bolded values are significantly different from the contralateral groups ($p<0.05$). Contra: contralateral eye, FD: form deprivation, sGAG: sulfated Glycosaminoglycans, AU: Arbitrary units (ratio of pixel intensities).

**Group**	**Chondroitin-4-Sulfate** [AU]	**Dermatan Sulfate** [AU]
Contra-1 (4)	1.50±0.20	3.09±0.53
FD-1 (5)	**1.34±0.25**	3.09±0.89
Contra-3 (6)	1.68±0.25	3.11±0.85
FD-3 (6)	**1.39±0.27**	**2.31±0.56**
